# Molecular Characterization and Zoonotic Potential of *Cryptosporidium* spp. and *Enterocytozoon bieneusi* in Wild Rodents and Sympatric Livestock in Xinjiang, China

**DOI:** 10.1155/tbed/5702480

**Published:** 2026-04-16

**Authors:** Yuman Li, Xinyu Hu, Zhen Li, Haowen Dong, Zhongying Yuan, Gang Liu, Xu Wang, Fang Shi, Jianping Cao, Yanyan Jiang, Suwen Wang, Yuanzhi Wang

**Affiliations:** ^1^ National Institute of Parasitic Diseases, Chinese Center for Disease Control and Prevention (Chinese Center for Tropical Diseases Research), National Key Laboratory of Intelligent Tracking and Forecasting for Infectious Diseases, NHC Key Laboratory of Parasite and Vector Biology, World Health Organization Centre for Tropical Diseases, Shanghai, 200025, China; ^2^ Key Laboratory for Prevention and Control of Emerging Infectious Diseases and Public Health Security, The Xinjiang Production and Construction Corps, Shihezi, 832003, Xinjiang Uygur Autonomous Region, China; ^3^ School of Medicine, Shihezi University, Shihezi, 832003, Xinjiang Uygur Autonomous Region, China, shzu.edu.cn; ^4^ College of Life Sciences, Shihezi University, Shihezi, 832003, Xinjiang Uygur Autonomous Region, China, shzu.edu.cn

**Keywords:** *Cryptosporidium*, *Enterocytozoon bieneusi*, wildlife–livestock interface, Xinjiang, zoonotic

## Abstract

*Cryptosporidium* spp. and *Enterocytozoon bieneusi* are significant enteric pathogens that affect a wide range of animal hosts, posing potential zoonotic risks. Wildlife–livestock interfaces are increasingly recognized as key settings for pathogen spillover, yet molecular epidemiological data from Xinjiang, China, remain limited. In this study, 191 fecal samples were collected from wild rodents and sympatric artiodactyls on the northern slope of the Tianshan Mountains in Bortala Prefecture, Xinjiang. Nested PCR targeting the small subunit (SSU) rRNA gene of *Cryptosporidium* spp. and the internal transcribed spacer (ITS) region of *E. bieneusi* was performed, followed by sequencing and phylogenetic analyses. Selected *Cryptosporidium*‐positive samples were further characterized at the *actin* and *hsp70* genes. Overall, *Cryptosporidium* spp. and *E. bieneusi* were detected in 5.8% (11/191) and 4.2% (8/191) of samples, respectively. Four *Cryptosporidium* genotypes were identified, including chipmunk genotype V, ground squirrel genotype II, *C. rubeyi*, and *C. xiaoi*. Rodent‐adapted genotypes predominated in long‐tailed ground squirrels and gray marmots, whereas *C. xiaoi* was detected in yak. While SSU rRNA and *actin* genes showed consistent clustering for several isolates, discordant phylogenetic signals at the *hsp70* gene suggested the possibility of coinfections. Four *E. bieneusi* genotypes were identified, including the zoonotic genotypes A, BEB6, XJHT4, as well as a novel genotype, XJWR1. The detection of genotype A in both rodents and sheep suggests potential interspecies transmission occurring at shared grazing sites. These findings expand the molecular evidence on *Cryptosporidium* spp. and *E. bieneusi* in Xinjiang and highlight wildlife–livestock interfaces as potential hotspots for pathogen exchange in transboundary pastoral ecosystems.

## 1. Introduction

*Cryptosporidium* spp. and *Enterocytozoon bieneusi* are enteric pathogens of increasing concern at the human–animal–environment interface due to their broad host ranges and capacity for cross‐species transmission [1, 2]. Rather than being restricted to single host species, these pathogens circulate among humans, wildlife, and domestic animals, forming complex transmission networks in shared environments. In pastoral landscapes, small mammals such as rodents inhabit areas that overlap with livestock grazing zones and human activities, thereby serving as potential ecological bridges between wildlife reservoirs and domestic animal populations [3–6]. Likewise, artiodactyls—including free‐ranging livestock and wild ungulates—may contribute to pathogen maintenance and dissemination through extensive environmental contamination [4, 7]. The persistence of environmentally resistant oocysts and spores in soil and water facilitates indirect transmission and enhances the potential for cross‐species exposure [8, 9]. Although cryptosporidiosis has been repeatedly associated with waterborne and foodborne outbreaks worldwide [10], infections caused by *E. bieneusi* are rarely identified in outbreak investigations [11–13], suggesting that its public health impact may be underestimated in settings characterized by close human–animal contact.

Molecular epidemiological studies have revealed substantial genetic heterogeneity in both pathogens, providing important insights into host adaptation and the risk of zoonotic transmission. In *Cryptosporidium* spp., numerous species and genotypes have been identified across a wide range of hosts, with molecular markers enabling discrimination between host‐adapted lineages and those capable of infecting multiple host groups [14, 15]. While several taxa exhibit strong host specificity—particularly among rodent‐associated lineages—others have been detected in both animals and humans, highlighting their potential for zoonotic transmission. For example, ground squirrel genotypes I–III, which are found exclusively in ground squirrels [16], *C. microti* in voles [17], and *C. rubeyi* in tree squirrels and chipmunks [17]. A similar pattern has been observed in *E. bieneusi*, where genotypes cluster into phylogenetic groups that differ markedly in host range [7, 18]. Group 1 genotypes (such as D, A, and Type IV) are frequently associated with human infection and multiple animal hosts [7], while other groups predominantly consist of host‐adapted lineages with limited evidence of cross‐species transmission, such as BEB6 in Group 2 and XJHT4 in Group 15 [19, 20]. These patterns indicate that spillover risk is unevenly distributed across genetic lineages and is strongly influenced by ecological context and host interactions.

Xinjiang provides a unique setting in which to investigate these dynamics. Its distinctive geography and climate support diverse wild rodent communities, including dominant species such as the long‐tailed ground squirrel (*Urocitellus undulatus*) and the gray marmot (*Marmota baibacina*) [21]. These rodents commonly coexist with free‐ranging livestock and wild artiodactyls along the northern slopes of the Tianshan Mountains, creating wildlife–livestock interfaces conducive to pathogen exchange [22]. Despite the potential epidemiological significance of these interactions, molecular data on *Cryptosporidium* spp. and *E. bieneusi* in wild rodents and sympatric artiodactyls from this region remain limited. To address this gap, the present study investigated the occurrence and genetic diversity of both pathogens in wild rodents and coexisting artiodactyls in northern Xinjiang to assess their potential for cross‐species transmission and zoonotic risk at the wildlife–livestock–human interface.

## 2. Materials and Methods

### 2.1. Ethics Approval and Consent to Participate

The present study procedures were reviewed and approved by the Ethics Committee of the National Institute of Parasitic Diseases, Chinese Centre for Disease Control and Prevention, China (Approval Number IPD‐2022‐014). Trapping, handling, and euthanasia of rodents followed the requirements of the Chinese Laboratory Animal Administration Act (2017).

### 2.2. Sampling Site and Sample Collection

Field sampling was carried out between July 2022 and June 2023 in the mountain–grassland ecotone located on the northern slope of the Tianshan Mountains, Bortala Prefecture, Xinjiang, China (~44°15′N, 82°50′E). This area represents a typical temperate continental ecosystem, characterized by mid‐ to low‐elevation mountainous terrain gradually transitioning into grassland and steppe habitats. The region supports abundant populations of wild rodents and free‐ranging artiodactyls that share overlapping ecological niches. Sampling covered two seasons, with fecal samples collected in spring (March–May) exclusively from wild rodents, whereas samples collected in summer (June–August) included both rodents and free‐ranging artiodactyls, reflecting seasonal variation in host and habitat use. A total of 191 fecal samples were collected. Among them, 152 samples originated from wild rodents, including long‐tailed ground squirrels (*Urocitellus undulatus*, *n* = 84), gray marmots (*Marmota baibacina*, *n* = 57), great gerbils (*Rhombomys opimus*, *n* = 10), and midday jirds (*Meriones meridianus*, *n* = 1). All rodent species involved in this study are nonendangered. Live rodents were captured using snap traps placed in areas with visible signs of rodent activity and inspected at regular intervals. Captured animals were humanely euthanized by CO_2_ inhalation in accordance with approved ethical guidelines, and intestinal contents were aseptically collected from each individual.

The remaining 39 samples were obtained from free‐ranging artiodactyls during the summer grazing season (June–August), when livestock and wildlife co‐occur extensively in shared pastures. These samples included yaks (*Bos grunniens*, *n* = 3), sheep (*Ovis aries*, *n* = 19), Siberian ibexes (*Capra sibirica*, *n* = 2), cattle (*Bos taurus*, *n* = 6), and red deer (*Cervus elaphus*, *n* = 9). Fecal samples were collected immediately after defecation from animals grazing on the same pasture, without physical restraint or disturbance. All samples were transported to the laboratory in cooled containers within 24 h of collection and stored at −20°C until further processing.

### 2.3. Sample Processing and DNA Extraction

Before DNA extraction, fecal samples were subjected to preliminary processing to reduce inhibitors and particulate debris. Intestinal contents from rodents were washed twice with distilled water and concentrated by centrifugation at 1500 g for 10 min. Fecal material from artiodactyls was first passed through a sieve and then washed by centrifugation under the same conditions to remove coarse plant fibers. Approximately 200 mg of processed material from each sample was used for genomic DNA extraction with the QIAamp DNA Mini Kit (Qiagen, Hilden, Germany), following the manufacturer’s protocol. The extracted DNA was eluted and stored at −80°C before PCR assays.

### 2.4. *Cryptosporidium* spp. and *E. bieneusi* Genotyping

To detect *Cryptosporidium* spp. and *E. bieneusi*, each DNA sample was subjected to two parallel nested PCR assays. *Cryptosporidium* spp. were identified and genotyped by nested PCR amplification of an ~830 bp nucleotide fragment of the small subunit (SSU) rRNA [23], an ~1066 bp nucleotide fragment of the *actin* gene [24], and an ~1950 bp nucleotide fragment of the 70‐kDa heat‐shock protein (*hsp70*) gene [25], following previously described protocols. *C. xiaoi* identified in this study was further subtyped by PCR and sequence analysis of the 60‐kDa glycoprotein (*gp60*) gene [26]. *E. bieneusi* was identified and genotyped by nested PCR amplification of an ~390 bp fragment of the rRNA gene, composing 76 bp of the 3′ end of the SSU rRNA gene, 243 bp of the internal transcribed spacer (ITS) region, and 70 bp of the 5′ end of the large subunit (LSU) rRNA gene, as previously described [27]. Each PCR assay included a positive control (cattle‐derived *C. parvum* DNA for *Cryptosporidium* spp. and human‐derived genotype D DNA for *E. bieneusi*) and a negative control (DNase‐free water). All PCR reactions were performed using 2× TransTaq‐T PCR SuperMix (+ dye), and amplicons were separated on 1.5% agarose gels and visualized with GelStrain on a Gel Doc XR + Imaging System (Bio‐Rad, USA). All reagents were obtained from TransGen Biotech Co., Beijing, China.

### 2.5. DNA Sequencing and Analysis

Secondary PCR products of the expected size were sequenced using the corresponding secondary primers on an ABI Prism 3730 XL DNA Analyzer (Applied Biosystems, Foster City, CA) by Shanghai Saiheng Biotechnology Company Limited (Shanghai, China). Sequence accuracy was confirmed through bidirectional sequencing. Obtained sequences were aligned and compared with reference sequences from GenBank using BLAST (http://www.ncbi.nlm.nih.gov/blast/) and analyzed in MEGA 7 (http://www.megasoftware.net/) to determine *Cryptosporidium* species/genotypes and *E. bieneusi* genotypes. Novel sequences were verified by sequencing at least two additional PCR products from the same DNA preparations. *E. bieneusi* genotypes were assigned based solely on the 243 bp ITS region according to the established nomenclature system [28].

### 2.6. Phylogenetic Analysis

Phylogenetic relationships were inferred using maximum‐likelihood (ML) analysis in MEGA 7, with evolutionary distances calculated using the Tamura 3‐parameter model. Tree reliability was evaluated by bootstrapping with 1000 replicates. The representative sequences generated in this study were submitted to GenBank under the following Accession Numbers: *Cryptosporidium*—PV491251–PV491253 (SSU rRNA), PV505170–PV505171 (*actin*), and PV505172–PV505173 (*hsp70*); *E. bieneusi*—PQ414922 (ITS).

### 2.7. Statistical Analysis

Confidence intervals (95% CIs) were calculated using OpenEpi (http://www.openepi.com/Proportion/Proportion.htm) (accessed January 20, 2026). Statistical analyses were performed with SPSS 27.0 (IBM Corp., Armonk, NY, USA). Differences in occurrence rates of *Cryptosporidium* spp. and *E. bieneusi* among sympatric animal species were assessed using Pearson’s chi‐square (*χ* ^2^) test and Fisher’s exact test, with *p* < 0.05 considered statistically significant. This approach was chosen because the small overall sample sizes for certain host species and the low number of positive detections make multivariate analyses unreliable.

## 3. Results

### 3.1. Occurrence of *Cryptosporidium* spp. and *E. bieneusi*

A total of 191 fecal samples were collected from animals belonging to the orders Rodentia and Artiodactyla and screened for *Cryptosporidium* spp. and *E. bieneusi* by nested PCR targeting partial SSU rRNA and ITS regions, respectively. Overall, *Cryptosporidium* spp. and *E. bieneusi* were detected in 11 (5.8%, 11/191, 95% CI: 3.1%–9.8%) and eight (4.2%, 8/191, 95% CI: 2.0%–7.8%) samples, respectively, with no significant difference in occurrence between the two pathogens (*χ* ^2^ = 0.23, *p* = 0.629) (Table 1).

**Table 1:** Occurrence and distribution of *Cryptosporidium* species/genotypes and *E. bieneusi* genotypes in animals in the northern Tianshan Mountain region of Xinjiang.

Order	Animal species (Latin name)	*Cryptosporidium* species/genotypes				*E. bieneusi* genotype	
		Number of positive/total number of examined (%)	SSU rRNA gene (*n*)	*actin* gene (*n*)^a^	*hsp70* gene (*n*)^a^	Number of positive/total number of examined (%)	ITS gene (*n*)
Rodentia	Long‐tailed ground squirrel (*Urocitellus undulatus*)	9/84 (10.7)	Chipmunk genotype V (6); ground squirrel genotype II (2); *C. rubeyi* (1)	Chipmunk genotype V (3); ground squirrel genotype II (1)	*C. microti* (3); ground squirrel genotype II (2)	3/84 (3.6)	A (2); XJWR1 (1)
	Gray marmot (*Marmota baibacina*)	1/57 (1.8)	Chipmunk genotype V (1)	—	—	2/57 (3.5)	A (1); XJHT4 (1)
	Great gerbil (*Rhombomys opimus*)	0/10 (0)	—			0/10 (0)	—
	Midday jird (*Meriones meridianus*)	0/1 (0)	—			0/1 (0)	—
	Subtotal	10/152 (6.6)	Chipmunk genotype V (7); ground squirrel genotype II (2); *C. rubeyi* (1)	Chipmunk genotype V (3); ground squirrel genotype II (1)	*C. microti* (3); ground squirrel genotype II (2)	5/152 (3.3)	A (3); XJWR1 (1); XJHT4 (1)
Artiodactyla	Yak (*Bos grunniens*)	1/3 (33.3)	*C. xiaoi* (1)	—	—	0 (0/3)	—
	Sheep (*Ovis aries*)	0/19 (0)	—			3/19 (15.8)	BEB6 (2); A (1)
	Siberian ibex (*Capra sibirica*)	0/2 (0)	—			0/2 (0)	—
	Cattle (*Bos taurus*)	0/6 (0)	—			0/6 (0)	—
	Red deer (*Cervus elaphus*)	0/9 (0)	—			0/9 (0)	—
	Subtotal	1/39 (2.6)	*C. xiaoi* (1)	—	—	3/39 (7.7)	BEB6 (2); A (1)
Total		11/191 (5.8)	Chipmunk genotype V (7); ground squirrel genotype II (2); *C. rubeyi* (1); *C. xiaoi* (1)	Chipmunk genotype V (3); ground squirrel genotype II (1)	*C. microti* (3); ground squirrel genotype II (2)	8/191 (4.2)	A (4); BEB6 (2); XJWR1 (1); XJHT4 (1)

*Note*: The bars “—” denote no results of PCR amplification.

^a^Molecular analyses of the *actin* and *hsp70* genes were performed only on positive samples at the SSU rRNA gene.

With respect to season, the occurrence of *Cryptosporidium* spp. was slightly higher in summer (6.5%, 9/139, 95% CI: 3.2%–11.6%) than in spring (3.8%, 2/52, 95% CI: 0.7%–12.1%), although this difference was not statistically significant (*p* = 0.730) (Table 2). *E. bieneusi* was detected only in summer samples (5.8%, 8/139, 95% CI: 2.7%–10.6%), while no positive samples were identified in spring (0/52); however, this seasonal difference was also not statistically significant (*p* = 0.110) (Table 2).

**Table 2:** Occurrence and seasonal distribution of *Cryptosporidium* species/genotypes and *E. bieneusi* genotypes in animals in the northern Tianshan Mountain region of Xinjiang.

Season	Animal species (Latin name)	*Cryptosporidium* species/genotypes				*E. bieneusi* genotype	
		Number of positive/total number of examined (%)	SSU rRNA gene (*n*)	*actin* gene (*n*)^a^	*hsp70* gene (*n*)^a^	Number of positive/total number of examined (%)	ITS gene (*n*)
Spring	Long‐tailed ground squirrel (*Urocitellus undulatus*)	2/43 (4.7)	Chipmunk genotype V (2)	—	—	0/43 (0)	—
	Great gerbil (*Rhombomys opimus*)	0/9 (0)	—			0/9 (0)	—
	Subtotal	2/52 (3.8)	Chipmunk genotype V (2)	—	—	0/52 (0)	—
Summer	Long‐tailed ground squirrel (*Urocitellus undulatus*)	7/41 (17.1)	Chipmunk genotype V (4); ground squirrel genotype II (2); *C. rubeyi* (1)	Chipmunk genotype V (3); ground squirrel genotype II (1)	*C. microti* (3); ground squirrel genotype II (2)	3/41 (7.3)	A (2); XJWR1 (1)
	Gray marmot (*Marmota baibacina*)	1/57 (1.8)	Chipmunk genotype V (1)	—	*—*	2/57 (3.5)	A (1); XJHT4 (1)
	Great gerbil (*Rhombomys opimus*)	0/1 (0)	—			0/1 (0)	—
	Midday jird (*Meriones meridianus*)	0/1 (0)	—			0/1 (0) 0/3 (0)	—
	Yak (*Bos grunniens*)	1/3 (33.3)	*C. xiaoi* (1)	—	*—*	0/3 (0)	—
	Sheep (*Ovis aries*)	0/19 (0)	—			3/19 (15.8)	BEB6 (2); A (1)
	Siberian ibex (*Capra sibirica*)	0/2 (0)	—			0/2 (0)	—
	Cattle (*Bos taurus*)	0/6 (0)	—			0/6 (0)	—
	Red deer (*Cervus elaphus*)	0/9 (0)	—			0/9 (0)	—
	Subtotal	9/139 (6.5)	Chipmunk genotype V (5); ground squirrel genotype II (2); *C. rubeyi* (1); *C. xiaoi* (1)	Chipmunk genotype V (3); ground squirrel genotype II (1)	*C. microti* (3); ground squirrel genotype II (2)	8/139 (5.8)	A (4); BEB6 (2); XJWR1 (1); XJHT4 (1)
Total		11/191 (5.8)	Chipmunk genotype V (7); ground squirrel genotype II (2); *C. rubeyi* (1); *C. xiaoi* (1)	Chipmunk genotype V (3); ground squirrel genotype II (1)	*C. microti* (3); ground squirrel genotype II (2)	8/191 (4.2)	A (4); BEB6 (2);XJWR1 (1); XJHT4 (1)

*Note*: The bars “—” denote no results of PCR amplification.

^a^Molecular analyses of the *actin* and *hsp70* genes were performed only on positive samples at the SSU rRNA locus.

At the host species level, the highest detection rate of *Cryptosporidium* spp. was observed in yaks (33.3%, 1/3, 95% CI: 1.7%–86.8%), followed by long‐tailed ground squirrels (10.7%, 9/84, 95% CI: 5.4%–18.8%) and gray marmots (1.8%, 1/57, 95% CI: 0.1%–8.3%). For *E. bieneusi*, the highest occurrence was detected in sheep (15.8%, 3/19, 95% CI: 4.2%–37.2%), followed by long‐tailed ground squirrels (3.6%, 3/84, 95% CI: 0.9%–9.4%) and gray marmots (3.5%, 2/57, 95% CI: 0.6%–11.1%) (Table 1). Notably, one long‐tailed ground squirrel was found to harbor coinfection with both *Cryptosporidium* spp. and *E. bieneusi*.

### 3.2. Distribution and Molecular Characterization of *Cryptosporidium* spp.

A total of 11 *Cryptosporidium*‐positive samples were successfully amplified and sequenced at the SSU rRNA gene, revealing four species/genotypes: chipmunk genotype V (*n* = 7), ground squirrel genotype II (*n* = 2), *C. rubeyi* (*n* = 1), and *C. xiaoi* (*n* = 1) (Table 1). Chipmunk genotype V was the most prevalent, accounting for 63.6% (7/11) of the positive samples, and was detected in long‐tailed ground squirrels (*n* = 6) and one gray marmot. Ground squirrel genotype II and *C. rubeyi* were exclusively observed in long‐tailed ground squirrels, while *C. xiaoi* was detected in a yak.

**Figure 1:**
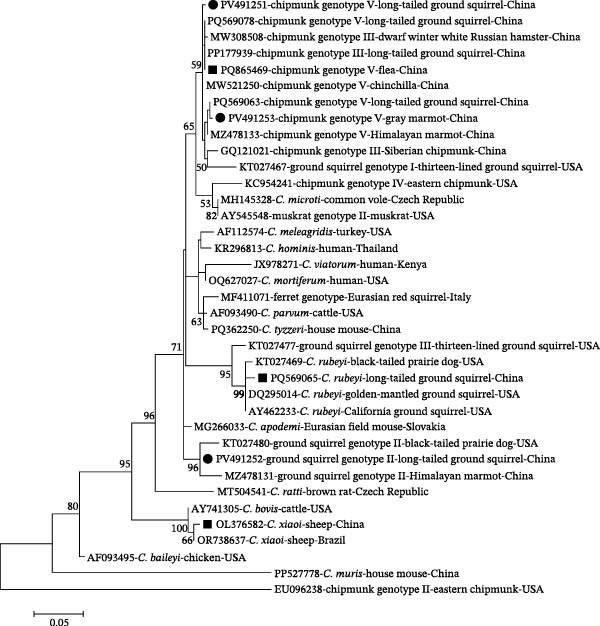
Phylogenetic relationships among various *Cryptosporidium* species/genotypes inferred by a maximum‐likelihood (ML) analysis of SSU rRNA gene sequences, based on genetic distances calculated by the Tamura 3‐parameter model. Numbers at the nodes represent the bootstrap values with more than 50% bootstrap support from 1000 replicates. The novel and known sequences generated in the present study are indicated with solid circles and squares, respectively.

Alignment of SSU rRNA sequences of chipmunk genotype V revealed three distinct sequence types. Five isolates from long‐tailed ground squirrels were identical to one another and shared 100% sequence identity with previously reported sequences from fleas (GenBank: PQ865469) and long‐tailed ground squirrels (GenBank: PP177939 and PQ569078) in China. The remaining two isolates—one from a long‐tailed ground squirrel and one from a gray marmot—were novel, showing 99.5% and 99.7% similarity with sequences from Himalayan marmots (GenBank: MZ478133 and PQ569063). The two ground squirrel genotype II isolates from long‐tailed ground squirrels were identical and shared 98.6% identity with Himalayan marmot isolates (GenBank: MZ478131). The *C. rubeyi* isolate from a long‐tailed ground squirrel was identical to a previously reported sequence (GenBank: PQ569065), and the *C. xiaoi* isolate from yak exhibited 100% identity with a sequence obtained from Tibetan sheep (GenBank: OL376582). Attempts to subtype *C. xiaoi* using the *gp60* gene were unsuccessful. Phylogenetic analysis of SSU rRNA sequences confirmed that all *Cryptosporidium* species and genotypes clustered with their respective reference sequences (Figure 1).

**Figure 2:**
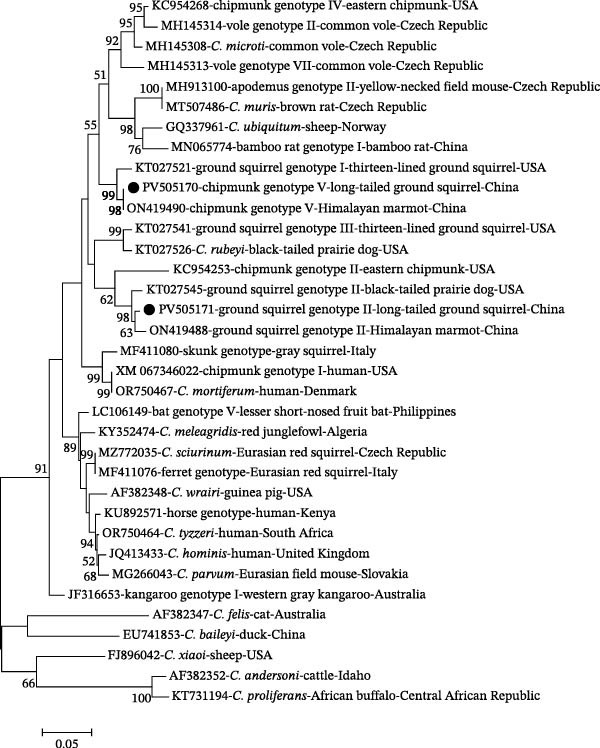
Phylogenetic relationships among various *Cryptosporidium* species/genotypes inferred by a maximum‐likelihood (ML) analysis of *actin* gene sequences, based on genetic distances calculated by the Tamura 3‐parameter model. Numbers at the nodes represent the bootstrap values with more than 50% bootstrap support from 1000 replicates. The sequences generated in the present study are indicated with solid circles.

**Figure 3:**
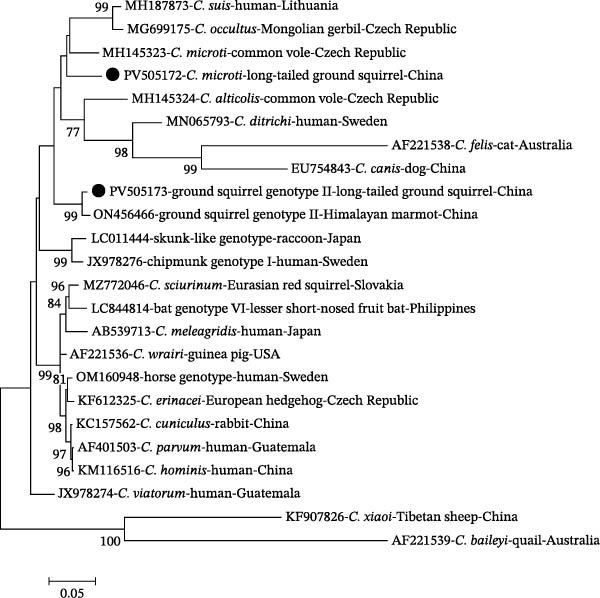
Phylogenetic relationships among various *Cryptosporidium* species/genotypes inferred by a maximum‐likelihood (ML) analysis of *hsp70* gene sequences, based on genetic distances calculated by the Tamura 3‐parameter model. Numbers at the nodes represent the bootstrap values with more than 50% bootstrap support from 1000 replicates. The sequences generated in the present study are indicated with solid circles.

To further assess genetic diversity, the 11 SSU rRNA‐positive DNA samples were analyzed at the *actin* and *hsp70* genes (Table 1). For chipmunk genotype V, three long‐tailed ground squirrel isolates were confirmed by *actin* sequencing, showing ≥99.8% similarity with Himalayan marmot isolates (GenBank: ON419490). In contrast, *hsp70* sequences from the same individuals displayed ~94.4% similarity to *C. microti* from common voles (GenBank: MH145323, Czech Republic), suggesting the presence of mixed infections. For ground squirrel genotype II, one *actin* sequence exhibited 98.3% similarity to a Himalayan marmot isolate (GenBank: ON419488), and two *hsp70* sequences were identical, showing 98.5% similarity to another Himalayan marmot isolate (GenBank: ON456466). Phylogenetic analyses at the *actin* (Figure 2) and *hsp70* genes (Figure 3) showed consistent clustering patterns, supporting both host‐adapted lineages and potential coinfections in long‐tailed ground squirrels.

### 3.3. Molecular Characterization of *E. bieneusi*

**Figure 4:**
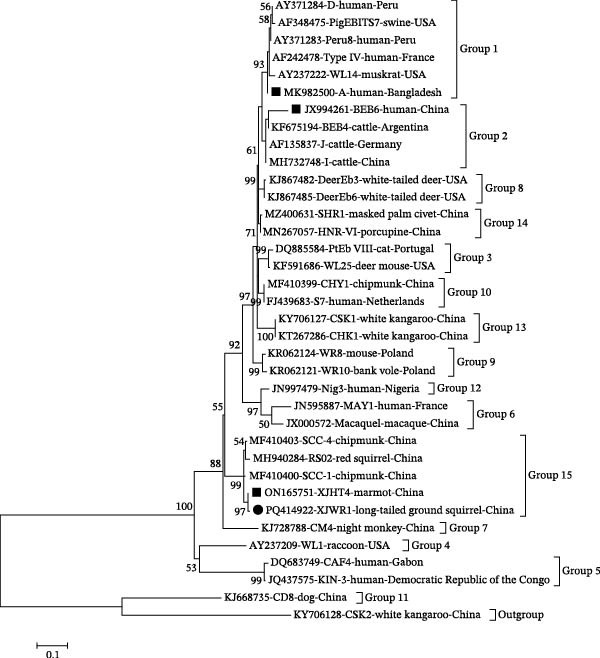
Phylogenetic relationships among various *E. bieneusi* genotypes inferred by a maximum‐likelihood (ML) analysis of ITS sequences, based on genetic distances calculated by the Tamura 3‐parameter model. Numbers at the nodes represent the bootstrap values with more than 50% bootstrap support from 1000 replicates. The novel and known genotypes are indicated with solid circles and squares, respectively.

Among the eight *E. bieneusi*‐positive samples, three previously reported genotypes—A (*n* = 4, 50.0%), BEB6 (*n* = 2, 25.0%), XJHT4 (*n* = 1, 12.5%)—and one novel genotype, XJWR1 (*n* = 1, 12.5%; GenBank: PQ414922), were identified (Table 1). The novel genotype XJWR1 differed from XJHT4 (GenBank: ON165751, China) by a single base substitution at position 68 (G→A) and shared 99.59% similarity. Phylogenetic analysis clustered the eight isolates into three groups (Figure 4). Group 1 comprised the zoonotic genotype A, including four isolates detected in long‐tailed ground squirrels (*n* = 2), a gray marmot (*n* = 1), and a sheep (*n* = 1). Group 2 consisted of the zoonotic genotype BEB6, with two isolates identified in sheep. The remaining two isolates belonged to Group 15, a rodent‐associated lineage, and included genotype XJHT4 from a gray marmot and the novel genotype XJWR1 from a long‐tailed ground squirrel (Figure 4).

## 4. Discussion

The results of this study provide molecular evidence for the circulation of *Cryptosporidium* spp. and *E. bieneusi* at the wildlife–livestock interface in Xinjiang, highlighting the complexity of transmission within the human–animal–environment interface. Rather than acting as isolated reservoirs, wild rodents and sympatric artiodactyls appear to contribute to overlapping transmission pathways that may facilitate pathogen persistence in pastoral ecosystems. Host availability and sample composition fluctuate seasonally, shaping these transmission dynamics. Wild rodents dominate as primary hosts in spring, whereas both rodents and free‐roaming artiodactyls contribute to the host assemblage during summer, increasing ecological overlap among species. Such seasonal convergence likely enhances opportunities for indirect transmission via environmental contamination, particularly through shared soil and water sources, thereby increasing exposure risks for both humans and livestock [29]. The detection of genetically diverse lineages in long‐tailed ground squirrels, with their presence in coexisting artiodactyls sharing the same habitats, underscores the importance of incorporating wildlife into surveillance frameworks aimed at assessing zoonotic risks in pastoral landscapes.

In the present study, *Cryptosporidium* spp. were detected in 5.8% of fecal samples collected from sympatric rodents and artiodactyls. Notably, a relatively high occurrence rate was observed in yaks (33.3%), representing the first molecular evidence of *Cryptosporidium* infection in this host species from Xinjiang. However, due to the limited sample size (*n* = 3), no reliable inference regarding the actual occurrence rate can be made. Previous studies from the Qinghai–Xizang Plateau have reported highly variable detection rates in yaks, ranging from 1.4% (8/577) in Tibet [30] to 54.4% (205/377) in Lhasa [31], highlighting substantial heterogeneity across regions and management systems. Although only a single positive sample was identified in the present study, yaks are free‐ranging animals that share grazing areas and water sources with other livestock and wildlife. Therefore, even sporadic *Cryptosporidium* infections may have epidemiological relevance, underscoring the need for larger‐scale surveys to clarify the prevalence and transmission role of yaks in this region. Among wild rodents, long‐tailed ground squirrels exhibited a detection rate (10.7%), closely matching previous findings from Urumqi [32], suggesting a relatively stable pattern of infection in this species across different localities within Xinjiang. *Cryptosporidium* was also identified in gray marmots, albeit at a low occurrence rate (1.8%). The detected species was consistent with host‐adapted lineages previously reported in marmots, in agreement with earlier observations from similar ecological settings [33]. Although the occurrence was low, gray marmots are long‐lived, wide‐ranging animals with burrowing behavior and repeated defecation in shared grazing areas, suggesting that even low‐level infections may contribute cumulatively to environmental contamination over time. In contrast, *Cryptosporidium* was not detected in several other sympatric species, including sheep, cattle, wild ungulates, and additional rodent species. This absence should also be interpreted cautiously, as limited sample sizes may have diminished the likelihood of detecting low‐intensity infections. Alternatively, it may reflect variations in exposure pathways, grazing behaviors, or habitat utilization between wildlife and domestic animals within extensive pastoral systems. Overall, the occurrence of *Cryptosporidium* spp. was lower in wild rodents (6.6%) and sympatric artiodactyls (2.6%) than that reported in farm‐associated settings, such as Madagascar (33.3% and 27.8%, respectively) [34], South Korea (13.3% and 20.9%, respectively) [35], and Ecuador (8.3% and 7.7%, respectively) [36]. These comparisons are not intended to imply direct equivalence, but rather to illustrate how transmission intensity may vary across ecological contexts characterized by differing host densities, levels of environmental contamination, and human or animal management practices.

Molecular characterization in this study revealed the presence of multiple *Cryptosporidium* species and genotypes, including *C. xiaoi* and several lineages generally considered rodent‐adapted, namely chipmunk genotype V, ground squirrel genotype II, *C. rubeyi*, and *C. microti*. Among these, *C. xiaoi* is of particular interest due to its broad host spectrum and documented occurrence in both domestic and wild animals. Initially described as a *C. bovis*‐like genotype and later formally recognized as a distinct species in 2009 [37], *C. xiaoi* is most frequently reported in small ruminants, especially sheep and goats [38], but has also been detected sporadically in cattle [39, 40], yaks [41, 42], muskoxen [43], deer [44, 45], fish [46], and kangaroos [47]. Although it is often regarded as predominantly host‐adapted with limited or uncertain zoonotic relevance [48], the detection of *C. xiaoi* in immunocompromised human patients suggests that its host range may not be entirely restricted [49]. Importantly, environmental evidence further supports the capacity of *C. xiaoi* to persist and disseminate beyond individual host species. Its identification in river water [50] and drinking water catchments [51] in various geographical regions suggests that aquatic environments can serve as secondary reservoirs. In agricultural settings, the concurrent detection of *C. xiaoi* in livestock feces and adjacent farm dams provides a plausible mechanism for indirect transmission and cross‐species exposure [52]. These findings collectively suggest that waterborne pathways may facilitate the circulation of *C. xiaoi* across livestock, wildlife, and potentially humans in shared landscapes.

**Figure 5:**
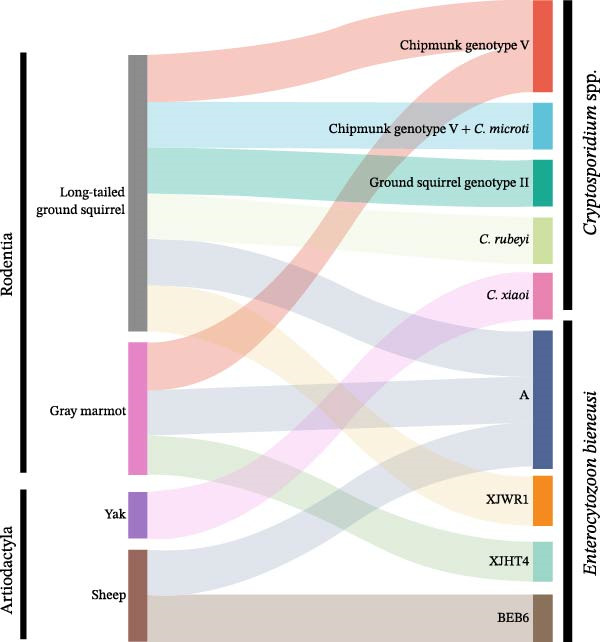
Host–pathogen flow diagram illustrating the distribution and transmission potential of *Cryptosporidium* species/genotypes and *E. bieneusi* genotypes among sympatric wildlife and livestock in Xinjiang.

In contrast, the remaining *Cryptosporidium* species and genotypes identified in this study—chipmunk genotype V, ground squirrel genotype II, *C. rubeyi*, and *C. microti*—have been reported exclusively in rodents, with no consistent evidence of infection in other mammalian orders to date. For example, chipmunk genotype V was first identified in chinchillas and chipmunks in China [53] and subsequently detected in Himalayan marmots [54]. Ground squirrel genotype II has thus far been confined to black‐tailed prairie dogs in the USA [16] and Himalayan marmots in China [54]. Similarly, *C. rubeyi* (formerly the *Cryptosporidium* c‐genotype) has been documented exclusively in wild rodents, including multiple ground squirrel species, prairie dogs, and Himalayan marmots [16, 54–57]. *C. microti*, a vole‐adapted species originally described in common voles [58], has since been reported in several rodent hosts, including field and root voles [59], red‐backed voles [32], and yellow‐necked mice [60], supporting its restriction largely to rodents. Notably, sequences closely related to *C. microti* were detected at the *hsp70* gene in samples assigned to chipmunk genotype V based on SSU rRNA and *actin* genes, suggesting possible coinfection in long‐tailed ground squirrels. Such coinfection may complicate genotype assignment and reflect more complex within‐host transmission dynamics, underscoring the need for confirmation using cloning or high‐throughput sequencing approaches. Collectively, these findings indicate that, unlike *C. xiaoi*, rodent‐adapted *Cryptosporidium* lineages are likely to be maintained primarily through intraorder transmission, with limited potential for cross‐order spillover. This pattern is further supported by phylogenetic clustering (Figure 1) and host distribution (Figure 5), which indicates circulation of closely related rodent‐adapted lineages among coexisting rodent hosts. Nonetheless, the coexistence of these lineages with *C. xiaoi* within the same ecological setting highlights the complexity of *Cryptosporidium* transmission networks and emphasizes the importance of both host specificity and environmental connectivity when assessing zoonotic risk. Our study further underscores the potential for cross‐species exposure at the wildlife–livestock interface, where rodent burrowing, repeated defecation, extensive grazing, and shared water sources may promote environmental contamination. Waterborne transmission of *C. xiaoi* is plausible, and although direct evidence is currently lacking, future environmental sampling and multilocus genotyping are warranted to clarify the relative contribution of these pathways to *Cryptosporidium* spp. circulation.

*E. bieneusi* was detected at an overall occurrence rate of 4.2% across all sampled hosts. In sheep, the occurrence rate (15.8%) was slightly below the global pooled estimate (17.4%) [61] and lower than those reported from several regions of China (21.8%–50.7%) [62–65] but exceeded rates observed in Portugal (2.2%) [66] and Turkey (8.0%) [67]. These findings are consistent with the widespread distribution of *E. bieneusi* in sheep populations worldwide and support the role of sheep as important reservoirs. In sympatric wild rodents, *E. bieneusi* was detected at relatively low occurrence rates in long‐tailed ground squirrels (3.6%) and gray marmots (3.5%), both of which were lower than those previously reported from Xinjiang [32, 68], suggesting spatial or temporal heterogeneity in transmission dynamics. No infections were detected in other sympatric hosts; however, this may be partly due to limited sample sizes, underscoring the need for more comprehensive investigations to determine the host range and prevalence. When analyzed by mammalian orders, the occurrence rates in wild rodents (3.3%) and cograzing artiodactyls (7.7%) were lower than those reported in farm‐associated rodents (4.0%) and cattle (11.9%) from Henan Province [22]. Differences in occurrence between wild and farm‐associated hosts may reflect ecological and management‐related factors, including host population density, degree of environmental contamination, and species‐specific susceptibility, which together may influence the intensity of *E. bieneusi* transmission.

Molecular characterization revealed four *E. bieneusi* genotypes in this study, encompassing zoonotic lineages (A and BEB6) with established public health relevance and rodent‐associated genotypes (XJHT4 and XJWR1). This genotype composition highlights a gradient of transmission risk at the wildlife–livestock interface. Among them, genotype A (Group 1) was the predominant genotype, accounting for 50.0% of all *E. bieneusi*‐positive samples and occurring in both sympatric wild rodents (long‐tailed ground squirrels and gray marmots) and sheep for the first time in this region. Genotype A, also known as Peru‐1 [69], is a well‐recognized human‐pathogenic genotype and has been reported in ~212 human cases worldwide [19, 70–73]. Beyond humans, it has been sporadically detected in a broad range of animal hosts, including nonhuman primates [74], dogs [75, 76], birds [77], brown rats [18], and cats [76]. Its detection in wastewater in Shanghai, China [78], further indicates that environmental contamination—particularly via water—may facilitate cross‐species transmission. The concurrent presence of genotype A in wildlife and livestock sharing the same habitats, as illustrated in Figure 5, provides molecular evidence for overlapping transmission pathways and underscores its zoonotic relevance in pastoral landscapes. Another zoonotic genotype detected in this study was BEB6 (Group 2). First described in cattle in the USA [79], this genotype was subsequently reported once in a child in Shanghai, China, under the name SH5 [80], though later studies have consistently used the original name BEB6 [19]. This genotype is now frequently detected in sheep worldwide [61], in agreement with our findings, and has also been reported in nine pediatric cases in China [80–82], providing further evidence of its zoonotic potential. In addition, the detection of BEB6 in wastewater from several Chinese cities [83, 84] and in raw milk from dairy cattle and sheep [85] suggests that both waterborne and foodborne pathways may contribute to human exposure, particularly in settings characterized by close contact between livestock, wildlife, and shared environmental resources.

In addition to the zoonotic genotypes, the rodent‐associated genotype XJHT4 was also identified in this study. This genotype has previously been reported in gray marmots [68], long‐tailed ground squirrels [86], and pygmy wood mice [86], and its detection in a gray marmot from this study further supports its association with sciurid rodents. Moreover, a novel genotype, designated XJWR1, was identified in a long‐tailed ground squirrel and differed from XJHT4 by a single nucleotide substitution at the ITS gene. Phylogenetic analysis placed both XJHT4 and XJWR1 within Group 15, clustering with other *E. bieneusi* genotypes derived from squirrels (order Rodentia, family Sciuridae), including SCC‐1, SCC‐4, and RS02, consistent with a rodent‐associated lineage. However, as XJWR1 is represented by a single isolate and is defined solely based on ITS variation, conclusions regarding its evolutionary divergence or host adaptation remain limited. Accordingly, although Group 15 genotypes appear to be host‐associated, their broader host range and zoonotic potential remain poorly defined. Continued surveillance, expanded sampling, and multilocus characterization are therefore warranted to clarify the transmission dynamics of Group 15 genotypes and to better assess their potential public health implications. Although Group 15 genotypes appear to be host‐associated, their broader host range and zoonotic potential remain poorly defined. In light of these uncertainties, continued surveillance and expanded sampling are warranted to clarify the transmission dynamics of Group 15 genotypes and to assess their potential public health implications.

Collectively, our findings indicate that rodent‐adapted *Cryptosporidium* lineages are mainly maintained through intraorder transmission, while *E. bieneusi* genotype A co‐occurs with livestock in shared habitats, highlighting the complexity of transmission networks at the wildlife–livestock interface. In pastoral areas, rodent burrowing and repeated defecation, combined with extensive grazing and reliance on natural water sources, may promote environmental contamination, creating plausible exposure scenarios for herders and livestock, although human infection data were not collected. Based on these findings, targeted surveillance is recommended in pastoral areas of Xinjiang, focusing on wild rodents, free‐ranging livestock, and shared water sources. Molecular monitoring of zoonotic genotypes using multilocus markers would improve tracking of cross‐species transmission. Intervention strategies should reduce environmental contamination and indirect contact, including improved management of communal grazing areas and water points, restricting livestock access to rodent‐active zones, and promoting safe water use among herders within a One Health framework.

## 5. Conclusion

This study provides the first molecular evidence of *Cryptosporidium* spp. and *E. bieneusi* in long‐tailed ground squirrels and sympatric animal species in Xinjiang, China, thereby expanding the known host range of both pathogens in this region. Multiple rodent‐adapted *Cryptosporidium* genotypes were identified in wild rodents, indicating predominantly host‐specific, intraorder transmission. In contrast, the detection of *C. xiaoi* and *E. bieneusi* genotypes A and BEB6 in wildlife and livestock highlights potential cross‐species exposure at the wildlife–livestock interface in pastoral ecosystems. Although the direct relevance of these two pathogens to human infection appears limited based on the current data, infected wild rodents and free‐ranging artiodactyls may contribute to environmental contamination of shared grazing areas and water sources, facilitating indirect transmission pathways. Therefore, these findings underscore the ecological complexity of pathogen circulation in coupled wildlife–livestock systems and underscore the importance of incorporating wildlife hosts into regional surveillance frameworks. Urgent research gaps include larger‐scale, systematic sampling across hosts, incorporation of environmental matrices such as water and soil, and multilocus or longitudinal approaches to better resolve transmission dynamics and refine zoonotic risk assessment within a One Health framework.

## Author Contributions

**Yuman Li and Xinyu Hu**: methodology, software, data curation, visualization, writing – original draft. **Zhen Li**, **Zhongying Yuan**, **Gang Liu**, and **Xu Wang**: methodology. **Haowen Dong and Fang Shi**: methodology, software. **Jianping Cao**: supervision. **Yanyan Jiang**: conceptualization, methodology, data curation, writing – review and editing, project administration. **Suwen Wang**: methodology, writing – review and editing. **Yuanzhi Wang**: conceptualization, project administration, writing – review and editing.

## Funding

This work was supported by the National Natural Science Foundation of China (Grant 82273693), the Science & Technology Innovation Team Project of TIANSHAN Elite (Grant 2023TSYCTD0020), the National Key Research and Development Program of China (Grant 2021YFC2300902), the Key Scientific & Technological Project of XPCC (Grant 2025AA018), the Key Laboratory for Prevention and Control of Emerging Infectious Diseases and Public Health Security, and the Xinjiang Production and Construction Corps (Grant EPH202301).

## Disclosure

The funding agencies had no role in the study design, data collection, analysis, and interpretation of data in the writing of the manuscript. The findings and conclusions of this study are those of the authors and do not necessarily represent the views of the funding agencies.

## Ethics Statement

This study protocol was approved by the Ethics Committee of the National Institute of Parasitic Diseases, Chinese Centre for Disease Control and Prevention, China (Approval Number IPD‐2022‐014). The capture and handling procedures of rodents complied with the Chinese Laboratory Animal Administration Act (2017).

## Conflicts of Interest

The authors declare no conflicts of interest.

## Data Availability

DNA sequences generated and analyzed in the current study are available in GenBank under Accession Numbers PV491251–PV491253 (SSU rRNA for *Cryptosporidium*), PV505170–PV505171 (*actin* for *Cryptosporidium*), and PV505172–PV505173 (*hsp70* for *Cryptosporidium*); PQ414922 (ITS for *E. bieneusi*).
